# Identification of predictive factors for surgical site infections in gastrointestinal surgeries: A retrospective cross-sectional study in a resource-limited setting

**DOI:** 10.12688/f1000research.135681.3

**Published:** 2024-05-30

**Authors:** Abdu Al-hajri, Saif Ghabisha, Faisal Ahmed, Saleh Al-wageeh, Mohamed Badheeb, Qasem Alyhari, Abdulfattah Altam, Afaf Alsharif

**Affiliations:** 1 Department of General Surgery, School of Medicine, Ibb University of Medical Sciences, Ibb, Yemen; 2 Department of Urology, School of Medicine, Ibb University of Medical Sciences, Ibb, Yemen; 3 Department of Internal Medicine, Faculty of Medicine, Hadhramaut University, Hadhramau, Yemen; 4 Department of General Surgery, School of Medicine, 21 September University, Sana'a, Yemen; 5 Department of Gynaecology, School of Medicine, Jeblah University for Medical and Health Sciences, Ibb, Yemen

**Keywords:** Surgical site infection, gastrointestinal surgery, predictors

## Abstract

**Background:**

Surgical site infection (SSI), albeit infrequent, drastically impacts the quality of care. This article endeavors to investigate the predictive factors of SSIs following surgical interventions that involve the gastrointestinal (GI) tract within a single institution in a resource-limited setting.

**Methods:**

Over seven years from June 2015 to June 2022, patients who underwent GI surgery and developed SSI were retrospectively matched with an unaffected case-control cohort of patients. Standardized techniques for wound culture, laboratory evaluation of bacterial isolates, and antibiotic susceptibility tests were employed. Logistic regression analysis was utilized to investigate the predictive factors associated with 30-day postoperative SSI occurrence.

**Results:**

A total of 525 patients who underwent GI surgical procedures were included, among whom, 86 (16.4%) developed SSI and the majority of SSIs were superficial (74.4%). Escherichia coli was the most commonly isolated bacterium (54.4%), and a high percentage of multidrug-resistant organisms were observed (63.8%). In multivariate Cox regression analysis, illiteracy (Odds ratio [OR]:40.31; 95% confidence interval [CI]: 9.54-170.26), smoking (OR: 21.15; 95% CI: 4.63-96.67), diabetes (OR: 5.07; 95% CI: 2.27-11.35), leukocytosis (OR: 2.62; 95% CI: 1.24-5.53), hypoalbuminemia (OR: 3.70; 95% CI: 1.35-10.16), contaminated and dirty wounds (OR: 6.51; 95% CI:1.62-26.09), longer operation duration (OR: 1.02; 95% CI: 1.01-1.03), emergency operations (OR: 12.58; 95% CI: 2.91-54.30), and extending antibiotic prophylaxis duration (OR: 3.01; 95% CI: 1.28-7.10) were the independent risk factors for SSI (all p < 0.05).

**Conclusions:**

This study highlights significant predictors of SSI, including illiteracy, smoking, diabetes, leukocytosis, hypoalbuminemia, contaminated and dirty wounds, longer operative time, emergency operations, and extending antibiotic prophylaxis duration. Identifying these risk factors can help surgeons adopt appropriate measures to reduce postoperative SSI and improve the quality of surgical care, especially in a resource-limited setting with no obvious and strict policy for reducing SSI.

## Introduction

A surgical site infection (SSI) is a frequently encountered nosocomial infection that typically develops within 30 days of surgery. In cases where an implant is used, the timeframe for SSI occurrence can extend up to one year. ^1^ The estimated incidence of SSI is 0.5% to 3% worldwide, with a higher incidence reported in low-income countries, where SSI is estimated to be the most common healthcare-associated infection. ^2^^,^^3^ In addition to the socioeconomic status, surgeries that involve the gastrointestinal (GI) appear to have a higher SSI incidence, with reports indicating a 12%-30% incidence rate of such cases. The associated expenditure of increased hospitalization (7-11 folds), mortality, and morbidity (2-11 folds) force a higher emphasis on detecting such patients earlier in the course of their illness and identifying patients with a higher risk of developing SSI to improve the quality of care and minimize the cost. ^4^^,^^5^

Various factors have been studied concerning SSI, which can extend from socioeconomic status to preoperative settings and surgical approaches. ^6^ Certain non-modifiable risk factors include age, gender, immunosuppression, diabetes mellitus, obesity, or active smoking. Additionally, the pre-operative preparation, operation duration, and intra-operative techniques may impact the development of SSI, which is seen at a higher rate in emergent and septic surgeries. ^3^^–^^5^ SSI can be attributed to microorganisms that are derived from the patient’s skin flora or the surrounding environment. ^1^ In either scenario, the adherence of microorganisms to the surgical instruments can contaminate the incision. Contaminated surgical procedures pose an increased risk, particularly when multidrug-resistant microorganisms are involved. ^6^

Previous monocentric and retrospective studies in Yemen reported SSI rates of 2.2% and 31.7%. ^7^^,^^8^ However, there is limited information available about the extent of SSI and its predictive factors in low-income countries, such as Yemen. ^7^^,^^8^ This study aimed to investigate the SSI rate and its predictive factors among Yemeni patients who underwent GI surgeries in a resource-limited setting.

## Methods

### Study design

A retrospective cross-sectional study was conducted to investigate the SSI rate in patients who underwent gastrointestinal surgery at Al-Thora Hospital, Ibb University, IBB, Yemen, between June 2015 and October 2022. We included 525 patients, from whom written informed consent was obtained. The study was approved by the Ethics Research Committees of Ibb University [ID: IBBUNI.AC.YEM.2023.75, on 03/03/2023].

### Inclusion criteria

Adult patients (≥18 years old), who had undergone either elective or emergency GI surgery at general surgery wards were included.

### Exclusion criteria

Exclusion criteria were pregnancy, anticoagulation, incomplete or concealing data, non-bowel-related surgeries ( *e.g.*, hernia), postoperative complications within more than 30 days of surgery, or admission to another hospital.

### Data collection

The study enrolled all eligible patients in consecutive order and utilized organized questionnaires to gather applicable information. This included demographics, including age, gender, educational level, body mass index (BMI), and place of residence, as well as health habits such as cigarette smoking and Khat chewing. In addition, comorbid conditions such as diabetes mellitus (DM), hypertension, chronic kidney, lung, and liver disease, history of malignancy, and preoperative blood transfusions were also documented. The American Society of Anesthesiologists (ASA) categorization system was used to measure preoperative physical state. Other information collected included the operative date, duration, wound nature, type, duration, anesthesia type, using the safety checklists, the urgency of surgery, readmission, reoperation, hair removal time, and details of preoperative antimicrobial administration (injection of amoxicillin and clavulanic acid 1.2 g for clean wounds and injection of ceftriaxone 1 g and metronidazole 500 mg for clean-contaminated wounds). Laboratory-collected data were white blood cell (WBC) counts, neutrophile percentage, and albumin levels.

The study documented surgery-related complications ( *e.g.*, SSI, fistula) in addition to non-surgical complications such as pneumonia, urinary tract infection (UTI), sepsis, and myocardial infarction (MI). Culture results and antibiotic sensitivities were also recorded, with wound swabs and pus specimens collected using standard microbiological techniques and transported to the laboratory for sensitivity analysis. Additionally, we collected the National Nosocomial Infections Surveillance (NNIS) index for each patient.

### Definitions

Based on the depth of infection, these SSIs were subsequently categorized into superficial (affecting the skin and subcutaneous tissue), deep (involving muscle and fascia), and organ space infections. ^6^ Wounds were classified into four categories depending on their level of contamination: clean, clean-contaminated, contaminated, or dirty-infected. The ASA score, which reflects the patient’s physical condition before the surgery, was determined through evaluation by the anesthesiologist using the ASA classification system. ^9^ The NNIS index considers three risk variables, each of which is worth one point: contaminated or dirty-infected surgical wound, ASA scores greater than 2, and operation length greater than T (where T is defined as the 75th percentile of the normal time for a surgical procedure). ^9^ The gastrointestinal cases were sorted into four categories (small bowel, large bowel, biliary, and pancreatic). ^10^ Leukocytosis was defined as a WBC count greater than 100 × 10 ^9^/L and hypoalbuminemia was defined as an albumin <3.5 g/dL.

### Study outcomes

The primary outcome was the prevalence of postoperative SSIs determined by assessing culture-positive results which were assessed by infection prevention and control staff diagnosis, according to the criteria set forth by the United States Center for Disease Control (CDC). This definition included infections affecting the superficial, deep, and organ space tissues of the surgical incision. The incidence of SSI was determined by evaluating and following up on all patients for 30 days following their surgery, by systematic visits, starting from the date of the operation. ^9^ It is important to note that medical complications such as pneumonia, MI, and UTI were separately documented and reported, and were not included in the definition of SSI or postoperative surgical complications. The secondary outcome was investigating the predictive factors for SSIs.

### Variables and measures

The outcome variable was SSI expressed as a binary variable: yes and no. Independent variables included Age (<60 years and ≥60 years), Sex (male and female), ASA score (Low [1 or 2] and High [3 or 4]), NNIS index (No risk, Low risk, Moderate risk, High risk), Surgical sites (Large bowel and Other gastrointestinal sites), Hospital stays (<5 days and ≥5 days), BMI (<30 kg/m ^2^ and ≥30 kg/m ^2^), Residency (Urban and Rural), Educational level (Educated and Illiterate), the Antibiotic time before surgery (<1 h and ≥1 h), hair removal time (<24 hours and ≥24 hours), WBC (<10×10 ^9^/L and ≥10×10 ^9^/L), Albumin (≥3.5 mg/dL and <3.5 mg/dL), Operative type (Elective and Emergency), Blood loss (<200 mL and ≥200 mL), Anesthesia type (Spinal and General), Wound class (I and II and III and IV), Temperature (<38°C and ≥38°C), and Operation duration (min). Additionally, Khat chewing, Smoking, History of hypertension, History of diabetes, History of chronic renal failure, History of chronic liver disease, History of lung disease, Perioperative blood transfusion, History of malignancy, Safety checklist used, and Drain insertion were presented as “yes” and “no”.

### Statistical analysis

IBM SPSS version 22 software (IBM Corp., Armonk, New York) was used for statistical analyses. Quantitative variables were presented as means and standard deviations, while qualitative variables were reported as frequencies and percentages. The normality of the data was confirmed using the Kolmogorov-Smirnov test. Statistical tests were used to compare qualitative and quantitative variables, including the independent samples t-test or Mann-Whitney test for quantitative variables, and the Chi-square or Fisher’s exact test for qualitative variables. All the continuous variables were converted into categorical variables for a better presentation of the nomogram.

Univariate analysis was conducted to identify the statistically significant variables associated with the development of SSIs. First, we did univariate logistic regression to determine the potential variables for SSI. When the P-value<0.05, the corresponding variable would be considered statistically significant. Second, Spearman’s rank correlation coefficient was used to analyze the correlations between statistically significant variables. If correlation coefficients>0.700 between different variables, the strongly correlated variables would be removed. To detect collinearity, the variance inflation factor (VIF) was calculated. If VIF>3.000 or tolerance<0.100, the corresponding variable would be removed. Next, we conducted a multivariate logistic regression analysis of all the statistically significant variables to examine their independence. The links between each risk factor and SSI were presented as an odds ratio (OR) and confidence interval (CI). A p-value of less than 0.05 was judged statistically significant. The ROC curve (receiver operating characteristic curve) was utilized to evaluate the risk adjustment prediction performance of the previous NNIS risk index and the Author’s model for post-gastrointestinal SSI, which contains the significant factors in multivariate analysis. ^9^

## Results

### Characteristics and presentation of patients

This study included a total of 525 patients, comprising 295 (56%) male patients and 230 (44%) female patients, with a mean age of 52.9±16.9. Table 1 provides a summary of the patients’ characteristics and presentation. The postoperative 30-day SSI occurred in 86 (16.4%) patients. A total of 193 (36.8%) of patients had ASA Class One. The operative case distribution was 206 (39.2%) in the small bowel, 182 (34.7%) in the large bowel, 124 (23.6%) in the biliary system, and 13 (2.5%) in the pancreatic system. The mean operation duration was 76.4±28.2 minutes. General complications were UTI and pneumonia in 5.5%, high-grade fever in 5.1%, and MI in 1% of patients. Laboratory and operative characteristics of patients are mentioned in Table 2.

**Table 1.** Patient characteristics of 525 patients who underwent gastrointestinal procedures.

Variables	N (%)
Age (year), Mean (SD)	52.2 (15.7)
**Sex**	
Male	295 (56.2)
Female	230 (43.8)
**Education level**	
Illiterate	200 (38.1)
Primary school	203 (38.7)
High school	122 (23.2)
**Residency**	
Urban	94 (17.9)
Rural	431 (82.1)
**Body mass index (kg/m** ^**2**^ **)**	
18.5-24.99	178 (33.9)
25-29.99	250 (47.6)
>30	97 (18.5)
**American Society of anesthesiologists’ class**	
1	193 (36.8)
2	178 (33.9)
3	123 (23.4)
4	31 (5.9)
Fever (Temperature ≥38°C)	251 (47.8)
Current smoking status	281 (53.5)
History of Hypertension	158 (30.1)
History of Diabetes	108 (20.6)
History of chronic renal disease	76 (14.5)
History of chronic liver disease	48 (9.1)
Hair removal time ≥24 hours of surgery	306 (58.3)
History of lung disease	26 (5.0)
History of malignancy	39 (7.4)
History of Khat chewing	425 (81.0)
**Operative type**	
Elective	202 (38.5)
Emergency	323 (61.5)
**Time of prophylaxis antibiotic injection**	
During 1 hour of operation	346 (65.9)
More than one hour of operation	179 (34.1)
**Operative case**	
Small bowel	206 (39.2)
Large bowel	182 (34.7)
Biliary	124 (23.6)
Pancreatic	13 (2.5)

**Table 2.** Laboratory and operative characteristics of 525 patients who underwent gastrointestinal procedures.

Variables	N (%)
Hypoalbuminemia (albumin <3.5)	69 (13.1)
Leukocytosis, Mean (SD)	24479 (48)
Neutrophil ≥85%	283 (53.9)
**Anesthesia type**	
Spinal	131 (25.0)
General	394 (75.0)
Safety checklists Used	471 (89.7)
**Wound calcification**	
Clean	197 (37.5)
Clean-contaminated	168 (32.0)
Contaminated	123 (23.4)
Dirty	37 (7.0)
**Blood loss**	
≥200 ml	102 (19.4)
<200 ml	423 (80.6)
Drain insertion	508 (96.8)
Operation duration (min), Mean (SD)	76.4 (28.2)
Hospital stays, (day), Mean (SD)	5.4 (1.7)
30-day postoperative surgical site infection	86(16.4)
**General complication**	
Urinary tract infections	29 (5.5)
Pneumonia	29 (5.5)
High-grade fever	27 (5.1)
Myocardial infarction	5 (1.0)

### Causative pathogens

Pathogens linked with SSI were identified from all SSI patient wounds. *Escherichia coli* (51.2%), *Enterococcus* spp. (17.4%), Bacteroides species (9.3%), and *Clostridium perfringens* (8.1%) were the most commonly isolated micro-organisms, with more than half of pathogenicity (63.8%) being multidrug-resistant organisms and the majority (70.1%) being extended-spectrum β-lactam producers ( Table 3). The majority of SSIs were superficial infections 64 (74.4%), while deep SSI infection was presented in 14 (16.3%), and organ-specific SSI infection was seen in 8 (9.3%) of cases.

**Table 3.** Distribution of pathogens identified in surgical site infections.

Culture result	N (%)	Hepatobiliary tract (%)	Large bowel tract (%)	Small bowel tract (%)
Escherichia coli	44 (51.2)	10 (40.0)	21 (67.7)	13 (43.3)
Enterococcus	15 (17.4)	4 (16.0)	5 (16.1)	6 (20.0)
Bacteroides species	8 (9.3)	4 (16.0)	2 (6.5)	2 (6.7)
Clostridium perfringens	7 (8.1)	2 (8.0)	3 (9.7)	2 (6.7)
Pseudomonas aeruginosa	5 (5.8)	2 (8.0)	0 (0.0)	3 (10.0)
Klebsiella	4 (4.7)	2 (8.0)	0 (0.0)	2 (6.7)
Anaerococcus prevotii	3 (3.5)	1 (4.0)	0 (0.0)	2 (6.7)

### The relationship between variables and SSI occurrences

The relationship between the independent factors and the dependent variable was explored using univariate and multivariate Cox regression analysis. On univariate analysis, Khat chowing, high ASA class (3 or 4), smoking, hypertension, diabetes, hypoalbuminemia, illiterate, contaminated and dirty wounds, higher temperatures ≥38°C, leukocytosis, neutrophile ≥85%, longer operation duration, blood loss more than 200 mL, biliary and pancreatic cases, longer hospital stay, hair removal ≥24 hours of surgery, presence of NNIS risk index, and emergency surgery were statistically significant associations with SSI occurrence (all p<0.05) ( Table 4).

**Table 4.** Univariate analysis of risk factors associated with surgical site infection.

Factors	Subgroup	No SSI (n = 439)	SSI (n = 86)	OR (95%CI)	P value
Sex	Male	249 (84.4)	46 (15.6)	Reference group	0.581
	Female	190 (82.6)	40 (17.4)	1.14 (0.71-1.81)	
Age groups	<60 years	299 (84.5)	55 (15.5)	Reference group	0.452
	≥60 years	140 (81.9)	31 (18.1)	1.20 (0.74-1.94)	
BMI (kg/m ^2^)	<30	359 (83.9)	69 (16.1)	Reference group	0.736
	≥30	80 (82.5)	17 (17.5)	1.11 (0.60-1.94)	
Residency	Urban	80 (85.1)	14 (14.9)	Reference group	0.667
	Rural	359 (83.3)	72 (16.7)	1.15 (0.63-2.21)	
Educational level	Educated	296 (91.1)	29 (8.9)	Reference group	**<0.001**
	Illiterate	143 (71.5)	57 (28.5)	4.07 (2.51-6.71)	
Khat chewing	No	94 (94.0)	6 (6.0)	Reference group	**0.003**
	Yes	345 (81.2)	80 (18.8)	3.63 (1.66-9.57)	
Smoking	No	240 (98.4)	4 (1.6)	Reference group	**<0.001**
	Yes	199 (70.8)	82 (29.2)	24.72 (10.08-82.01)	
History of hypertension	No	286 (77.9)	81 (22.1)	Reference group	**<0.001**
	Yes	153 (96.8)	5 (3.2)	0.12 (0.04-0.26)	
History of diabetes	No	373 (89.4)	44 (10.6)	Reference group	**<0.001**
	Yes	66 (61.1)	42 (38.9)	5.39 (3.28-8.89)	
History of chronic renal failure	No	379 (84.4)	70 (15.6)	Reference group	0.236
	Yes	60 (78.9)	16 (21.1)	1.44 (0.77-2.60)	
History of chronic liver disease	No	399 (83.6)	78 (16.4)	Reference group	0.955
	Yes	40 (83.3)	8 (16.7)	1.02 (0.43-2.16)	
History of lung disease	No	417 (83.6)	82 (16.4)	Reference group	0.888
	Yes	22 (84.6)	4 (15.4)	0.92 (0.27-2.49)	
Antibiotic time before surgery	<1 h	292 (84.4)	54 (15.6)	Reference group	0.506
	≥1 h	147 (82.1)	32 (17.9)	1.18 (0.72-1.89)	
Perioperative blood transfusion	No	408 (83.4)	81 (16.6)	Reference group	0.676
	Yes	31 (86.1)	5 (13.9)	0.81 (0.27-1.98)	
Hair removal time before surgery	<24 h	199 (90.9)	20 (9.1)	Reference group	**<0.001**
	≥24 h	240 (78.4)	66 (21.6)	2.74 (1.63-4.77)	
History of malignancy	No	405 (83.3)	81 (16.7)	Reference group	0.534
	Yes	34 (87.2)	5 (12.8)	0.74 (0.25-1.78)	
Temperature	<38°C	248 (90.5)	26 (9.5)	Reference group	**<0.001**
	≥38°C	191 (76.1)	60 (23.9)	3.00 (1.84-5.00)	
WBC (10 ^9^/L)	<10×10 ^3^	221 (90.6)	23 (9.4)	Reference group	**<0.001**
	≥10×10 ^3^	218 (77.6)	63 (22.4)	2.78 (1.69-4.72)	
Albumin	≥3.5mg/dL	397 (87.1)	59 (12.9)	Reference group	**<0.001**
	<3.5mg/dL	42 (60.9)	27 (39.1)	4.33 (2.47-7.52)	
Operative type	Elective	172 (85.1)	30 (14.9)	Reference group	**<0.001**
	Emergency	267 (82.7)	56 (17.3)	1.20 (0.75-1.97)	
Wound class	I and II	329 (90.1)	36 (9.9)	Reference group	**<0.001**
	III and IV	110 (68.8)	50 (31.2)	4.15 (2.58-6.75)	
Anesthesia type	Spinal	110 (84.0)	21 (16.0)	Reference group	0.900
	General	329 (83.5)	65 (16.5)	1.03 (0.61-1.81)	
Safety checklist used	Yes	395 (83.9)	76 (16.1)	Reference group	0.654
	No	44 (81.5)	10 (18.5)	1.18 (0.54-2.36)	
Blood loss	<200 ml	96 (94.1)	6 (5.9)	Reference group	**0.003**
	≥200 ml	343 (81.1)	80 (18.9)	3.73 (1.71-9.83)	
Operation duration (min)	Mean (SD)	72.5 (25.9)	96.5 (30.7)	1.03 (1.02-1.04)	**<0.001**
Drain insertion	No	15 (88.2)	2 (11.8)	Reference group	0.603
	Yes	424 (83.5)	84 (16.5)	1.49 (0.41-9.54)	
Hospital stays	<5 days	177 (92.2)	15 (7.8)	Reference group	**<0.001**
	≥5 days	262 (78.7)	71 (21.3)	3.20 (1.82-5.96)	
Surgical site	Large bowel	151 (83.0)	31 (17.0)	Reference group	0.769
	Small bowel and other GI sites	288 (84.0)	55 (16.0)	1.08 (0.66-1.73)	
NNIS index	No risk	240 (96.0)	10 (4.0)	Reference group	
	Low risk	86 (76.8)	26 (23.2)	7.26 (3.46-16.37)	**<0.001**
	Moderate risk	87 (68.0)	41 (32.0)	11.31 (5.64-24.79)	**<0.001**
	High risk	26 (74.3)	9 (25.7)	8.31 (3.05-22.51)	**<0.001**
ASA score	Low (1 or 2)	322 (86.8)	49 (13.2)	Reference group	**0.003**
	High (3 or 4)	117 (76.0)	37 (24.0)	2.08 (1.29-3.34)	

**Abbreviations:** BMI: Body mass index, WBC: white blood cells, ASA scores: American Society of Anesthesiology, NNIS index: National Nosocomial Infections Surveillance, CI: confidence interval, OR: odds ratio, SSI: surgical site infection.

### Multivariate logistic regression revealed the following independent risk factors

**Figure 1.**
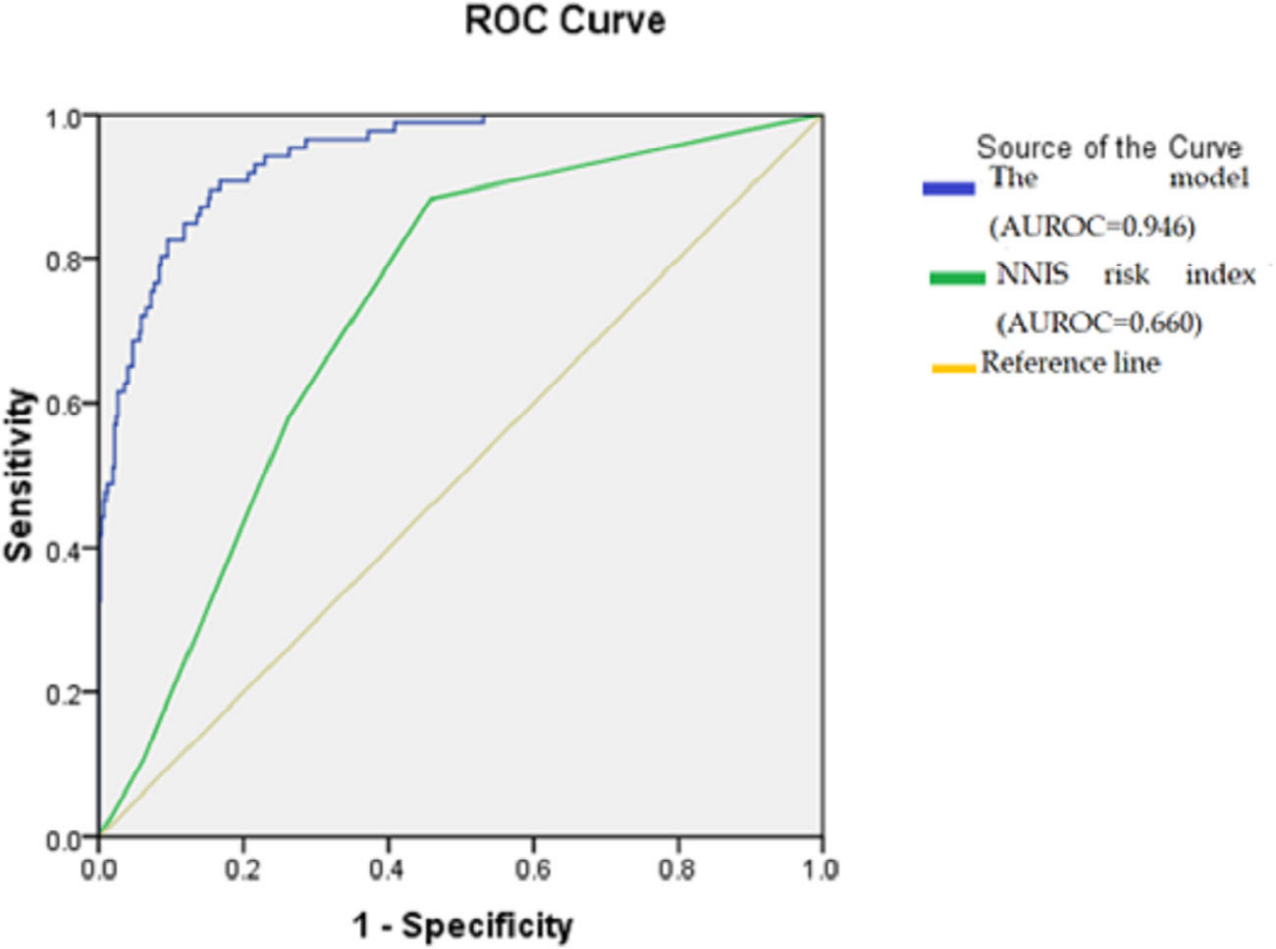
The receiver operating characteristic curve of the prediction model compared with the National Nosocomial Infections Surveillance risk index in the validation cohort. Abbreviations: AUROC: Area under the receiver operating characteristic curve; NNIS: National Nosocomial Infections Surveillance.

Illiteracy (OR: 40.31; 95% CI: 9.54-170.26), current smoking (OR: 21.15; 95% CI: 4.63-96.67), diabetes (OR: 5.07; 95% CI: 2.27-11.35), leukocytosis (OR: 2.62; 95% CI: 1.24-5.53), hypoalbuminemia (OR: 3.70; 95% CI: 1.35-10.16), contaminated and dirty wounds (OR: 6.51; 95% CI: 1.62-26.09), longer operative duration (OR: 1.02; 95% CI: 1.01-1.03), emergency operations (OR: 12.58; 95% CI: 2.91-54.30), and administering antibiotics before 1 hour of operation (OR: 3.01; 95% CI: 1.28-7.10) were independent factors for SSI (all p-value<0.05, Table 5). The prediction model’s total ROC curve was 0.946, which was much higher than the NNIS score (0.660) ( Figure 1).

**Table 5.** Multivariate analysis of risk factors associated with surgical site infection.

Predictor	Estimate	SE	Z	P value	OR	95%CI	
						Lower	Upper
Education level	3.6966	0.73508	5.0288	**<0.001**	40.31	9.54351	170.26
Khat chewing	0.6876	2.97657	0.2310	0.817	1.99	0.00582	679.66
Smoking	3.0518	0.77527	3.9365	**<0.001**	21.15	4.62903	96.67
Hypertension	-0.8014	0.84596	-0.9473	0.343	0.45	0.08548	2.36
Diabetes	1.6240	0.41073	3.9539	**<0.001**	5.07	2.26816	11.35
Hair removal time	0.5350	0.41789	1.2802	0.200	1.70	0.75272	3.87
Temperature	0.2583	0.39358	0.6563	0.512	1.29	0.59864	2.80
leukocytosis	0.9643	0.38089	2.5316	**0.011**	2.62	1.24327	5.53
Hospital stays	0.2963	0.47340	0.6260	0.531	1.34	0.53180	3.40
Albumin	1.3094	0.51494	2.5429	**0.011**	3.70	1.35007	10.16
Wound class	1.8735	0.70827	2.6451	**0.008**	6.51	1.62461	26.09
Blood loss	0.6588	2.99194	0.2202	0.826	1.93	0.00549	680.54
Operation duration	0.0214	0.00644	3.3313	**<0.001**	1.02	1.00886	1.03
ASA score	-0.2291	0.58612	-0.3909	0.696	0.79	0.25212	2.51
NNIS score	-0.0705	0.85520	-0.0825	0.934	0.93	0.17435	4.98
Operative type	2.5323	0.74601	3.3944	**<0.001**	12.58	2.91575	54.30
Antibiotic time	1.1032	0.43687	2.5253	**0.012**	3.01	1.28013	7.10
**Accuracy:** 0.905; **Specificity:** 0.966; **Sensitivity:** 0.593; **AUC:** 0.946. Nagelkerke R square: 0.648 Significance of the model <0.001							

**Abbreviations:** ASA scores: American Society of Anesthesiology, NNIS index: National Nosocomial Infections Surveillance, SE: standard error, CI: confidence interval, OR: odds ratio.

## Discussion

The improved access to healthcare, increased population age, and increased complexity of surgical interventions and patients’ conditions shed light on the importance of managing post-operative complications. Despite the precautions and the hygienic approach implemented to limit the incidence of SSI, it still represents one of the most common post-operative complications. Such infections result in an increased healthcare expenditure, and worsened mortality and morbidity. ^10^ This predicament can be especially disadvantageous for low-income nations, where providing healthcare is already a daunting task due to constrained resources, indigent communities, and elevated levels of antimicrobial resistance. ^11^

Among the 525 enrolled patients, the incidence of SSI within 30 days after surgery was 16.4%, which is in line with previous reports from developing countries, such as Saudi Arabia, with a rate of 16.3%. ^14^ However, earlier studies showed much higher rates of SSI affecting up to one-third of the patients in Yemen. ^8^ In contrast, more recent reports from Yemen have demonstrated a lower incidence of SSI, with a rate of 12.7% among patients who underwent gastrointestinal procedures. ^8^ Our findings, which showed a slightly higher rate of SSI, could be partially attributed to the larger number of complicated cases or complex oncological procedures performed at our tertiary teaching hospital.

Several studies have been conducted to evaluate the link between putative risk variables and SSI in GI surgical operations. However, there is a large range of variation in the variables analyzed and the proportional effect of these factors on individual outcomes. To address this issue, we comprehensively studied the preoperative and operational risk variables in GI operations associated with the development of postoperative SSI. Hamza *et al*. and Lakoh *et al*. carried out similar investigations. ^6^^,^^12^ This study found that illiteracy, current smoking status, DM, leukocytosis, hypoalbuminemia, contaminated and dirty wounds, longer operation duration, emergency operations, and longer time between administering antibiotics and operation were predictors for the development of SSI. Most of the potential predictive factors included have been previously reported as risk factors in other studies with a variety of reports and different levels. ^6^^,^^12^

The relationship between age and SSI risk is complex and not well understood. While some studies have reported an increased rate of infection in older patients, others have observed a favorable trend with increasing age. For instance, Kaye *et al*. demonstrated a 1.2% decrease in SSI risk for each additional year after 65 years of age. ^13^ Nevertheless, these findings were demonstrated consistently, as a higher rate of SSI was observed in the older population. ^14^^,^^15^ Typically, with increasing age, there is an accumulated risk of developing comorbidities and immune dysfunction, which may lead to an increased likelihood of SSI. However, our study’s findings revealed no association between age and the development of SSI. This divergence may be attributed to variations in age categorization, as the majority of patients (67%) in this study were younger than 65 years.

The present study reveals a significant association between the level of literacy and the incidence of SSI. Specifically, illiterate patients were 40 times more susceptible to SSIs than educated patients. These results are in accordance with previous research conducted by Mezemir *et al*. and Baker *et al.* ^16^^,^^17^ Notably, a high prevalence of limited health literacy among adults in our country may adversely impact health outcomes. For example, patients with limited health literacy may experience difficulty in comprehending complex health information, may exhibit non-compliance with postoperative instructions, and may not adequately prepare for surgery. These factors may increase the risk of SSIs and other adverse outcomes, highlighting the potential health inequality in providing care and education for illiterate patients. Therefore, it is critical to improve health literacy among patients, particularly those with limited education, to potentially reduce the incidence of SSIs and enhance surgical outcomes.

This study did not find a significant association between unmodifiable risk factors, such as gender, BMI, residency, number of comorbidities (hypertension, history of malignancy, CRF, liver and lung diseases), perioperative blood transfusion, and SSIs in multivariate analysis. Although these social determinants are important factors that may contribute to patient outcomes, there is a lack of consensus on their association with SSI occurrence in the literature. For example, Marzoug *et al.* found that male sex and a greater number of comorbidities were associated with SSI occurrence. ^18^ Additionally, Li *et al*. reported that ascites, bleeding diathesis, history of lung disease, radiotherapy, chemotherapy, chronic steroid use, and weight loss were associated with SSI occurrence. ^19^ In contrast, Mezemir *et al*. did not find an association between gender, BMI, and SSI occurrence, which was similar to our study. ^16^ These discrepancies may be attributed to sample size and demographics variation across studies, as well as variations in the documentation and management of patient comorbidities. The use of more objective measures, such as preoperative laboratory and radiologic values, may provide a better understanding of the association between comorbidities and SSI occurrence. Distinctly, in this study, DM and hypoalbuminemia had 5- and 3.7 times higher chances of developing SSIs, respectively. This association was observed in prior studies, ^11^^,^^20^ as hyperglycemia has been shown to impair WBC functions, leading to decreased immunity. ^21^ On the other hand, reduced serum albumin levels are often associated with malnutrition or chronic wasting diseases. ^11^ However, the glucose levels were not available for the included patients and the albumin level was converted into categorical variables for a better presentation of the nomogram.

Our study revealed that smoking was strongly associated with a 21-fold increased risk of developing SSIs compared to non-smokers. The vasoconstrictive and toxic effects of smoking are known to impede tissue oxygen delivery and hinder the healing process, thus contributing to the development of SSIs. These findings align with previous reports by Mawalla *et al*. and Billoro *et al*. ^22^^,^^23^

Regarding Khat ( *Catha edulis*) chewing, its role in SSI occurrence remains uncertain. Our study observed a 1.99-fold increase in SSI occurrence among Khat chewers, although this association was not statistically significant. Currently, there is a lack of published studies specifically investigating the relationship between SSI and Khat chewing. However, Misha *et al*. found no association between Khat chewing and SSI occurrence in their regression analysis. ^3^ Nevertheless, Khat chewing has been linked to various gastric issues ( *e.g.*, intestinal obstruction, and gastritis). ^24^ Furthermore, long-term Khat consumption poses a risk of developing severe complications including hepatitis, hepatic fibrosis, and cirrhosis in advanced stages. ^25^ Future prospective and more inclusive studies are recommended to investigate this issue, particularly in our country where the traditional use of these plants is widespread.

The settings of operation can significantly impact the development of SSI. Prior research has suggested that the degree of intraoperative wound contamination is indicative of SSI occurrence. ^26^^,^^27^ We found that contaminated and dirty wounds were 6.51 times more likely to develop SSI, which was consistent with other studies. ^26^^–^^28^ In this study, no statistically significant difference in SSI occurrence between colorectal procedures and other GI site procedures. However, most large-bowel SSIs were deep SSI types (8/31 in large bowel procedures vs., 3/25 in biliary and pancreatic procedures vs., 3/30 in small bowel procedures). According to data published by the National Healthcare Safety Network, rates of SSI following bile duct, liver, or pancreatic surgery are as high as 10 per 100 procedures. Rates of SSI following colon surgery are approximately 5 per 100 procedures, and rates of SSI following gallbladder surgery are 0.7 per 100 procedures. ^29^ Bozzay *et al.* study, the incisional SSI rates were higher following small bowel and gastrostomy closure procedures than for colorectal procedures and 66.1% of the cumulative incisional SSI burden from all procedures was attributable to 3 procedure groups (gastrostomy: 27.5%, small bowel: 22.9%, colorectal: 15.7%). ^30^

However, our findings were inconsistent with the literature documenting pancreatic and biliary leaks as independent risk factors for SSI occurrence. ^27^ This discrepancy could be attributed to the low number of cases involving biliary and pancreatic procedures, with most of them undergoing simple operations. Therefore, further prospective studies with a larger number of cases are necessary to clarify this issue.

Our study also found that emergency operations were 12.58 times more likely to result in SSI, consistent with other studies. ^6^^,^^11^ In addition, leukocytosis was found to be a predictor for the development of SSI, which aligns with previous research. ^31^ Additionally, prolonged operation duration was recognized as an independent factor for SSI development in other studies, as it increases the risk of infection due to extensive surgical procedures and incisions, prolonged anesthesia, blood loss, and weaning antimicrobial prophylaxis concentration. ^6^^,^^32^ Furthermore, administering antibiotics one hour before operation has been reported as a predictor for SSI in previous studies. ^23^^,^^33^ In this study, it was observed that longer operation durations and administration of antibiotics more than one hour before the operation increased the likelihood of SSI by 1.02 times and 3.01 times, respectively. These findings are consistent with previous studies that showed the importance of re-dosing when this duration reaches the half-life of the administered antibiotic and guidelines recommend it. ^23^^,^^32^

In this study, the time for hair removal was not statistically significant in multivariate analysis. This was in line with a recently published systematic review by Tanner et al. who mentioned that hair removal with clippers or depilatory cream may reduce the risk of SSIs, but not fewer than shaving with a razor. Moderate-certainty evidence suggests clippers or creams may reduce SSIs and complications. Hair removal on the day of surgery may also reduce risk. ^33^

On the other hand, Zhang *et al.* reported that patients undergoing hair removal (the day of surgery or the night before surgery) had lower SSI incidence compared with those without hair removal. ^34^

In this study, we investigated the microorganisms responsible for SSIs and their susceptibility to commonly prescribed prophylactic antibiotics. We found that the most common organisms isolated from infected wounds were Gram-negative bacteria, with extended-spectrum β-lactamase-producing *E. coli* being the most prevalent. Mawalla *et al*. reported a different outcome compared to this finding, as their studies indicated a higher presence of Gram-positive bacteria, including *Staphylococcus aureus.* ^22^ In contrast, studies have reported similar findings to ours, demonstrating a higher occurrence of Gram-negative bacteria in infected abdominal wounds. ^26^^,^^35^ Furthermore, our findings revealed a high prevalence of multi-resistant pathogens in relation to commonly prescribed prophylactic antibiotics, which may serve as an explanation for the elevated rate of deep SSI observed in our study. Hence, there is a need to consider appropriate prophylactic antibiotics, especially for high-risk patients. Additionally, strict adherence to surgical site infection prevention techniques Such as disinfection and sterilization of medical and surgical tools to avoid the spread of infectious germs need to get more attention. Healthcare rules should specify whether cleaning, disinfection, or sterilization is required based on the item's intended usage.

The NNIS risk index is a widely recognized framework for assessing and predicting the likelihood of SSI. ^9^ Within our study, two elements of the NNIS exhibited statistical significance (operation duration and wound class). However, upon conducting multivariate analysis, the overall NNIS model did not yield statistical significance. Moreover, when comparing the predictive accuracy, our developed model outperformed the NNIS model. Acutely, the performance of the NNIS model in this study showed poor predictive performance for the SSI occurrence as determined by the ROC curve. These results align with previous findings reported by Zhang *et al*. ^11^

Surgical site infections can be prevented by a variety of techniques, including improved preoperative surgical site preparation, good infection control management during procedures, careful adherence to prophylactic antibiotics administration, and a variety of preventive measures aimed at neutralizing the threat of bacterial, viral, and fungal contamination posed by operative staff, the operating room environment, and the patient's endogenous skin flora. Glucose-level control, improved oxygen supply, and normothermia maintenance are three new areas that have the potential to lower the incidence of SSIs even further. Continuous study into the biology of SSIs, as well as rigorous adherence to the use of evidence-based proven techniques to minimize SSIs, can help to further reduce the health and cost repercussions of SSIs. ^6^^,^^12^

### Study limitations

There are several limitations to consider in this study. First, the retrospective nature of the study may introduce an unintended bias to the study. In addition, it was conducted at a single tertiary teaching hospital, which may limit the generalizability of the findings to other healthcare settings. Furthermore, the study relied on clinical documentation to identify SSI, which could lead to underreporting or misclassification of cases. Moreover, the study focused on a specific geographic region, and the findings may not apply to other populations with different demographics or healthcare systems. Although the study took into account certain potential confounding variables ( *e.g.*, the use of prophylactic antibiotics), other potential confounding variables are difficult to assess with the retrospective nature of the study ( *e.g.*, surgical techniques and intra-operative maintenance of sterile technique, among others). Finally, the study did not explore long-term outcomes or evaluate the impact of interventions aimed at reducing surgical site infections. Based on our findings, further research needs to be validated in a large prospective cohort study with a long-term post-procedural follow up and the use of local coordinators may minimize these potential biases.

## Conclusions

This study highlights significant predictors of SSI, including illiteracy, active smoking, DM, leukocytosis, hypoalbuminemia, contaminated and dirty wounds, longer operation duration, emergency operations, and extending antibiotic prophylaxis duration. *Escherichia coli* was the most common pathogen and had a high rate of multidrug-resistant strains. Identifying these risk factors can help surgeons adopt appropriate measures to reduce SSI and improve the quality of surgical care, especially in a resource-limited setting with no obvious and strict policy for reducing SSI.

### Ethical considerations

Ethical approval was granted by the Ethics Research Committees of Ibb University [ID: IBBUNI.AC.YEM.2023.75, on 03/03/2023].

## Data Availability

Mendeley Data: Identification of Predictive Factors for Surgical Site Infections in Gastrointestinal Surgeries: A Retrospective Cross-Sectional Study in a Resource-Limited Setting, http://dx.doi.org/10.17632/hk75wrwr6n.1. ^36^ Data are available under the terms of the Creative Commons Attribution 4.0 International license (CC-BY 4.0).
