# Supplementary material for: Identification of predictive factors for surgical site infections in gastrointestinal surgeries: A retrospective cross-sectional study in a resource-limited setting
Source: F1000Res. 2024 May 30;12:733. Originally published 2023 Jun 23. [Version 3] doi: 10.12688/f1000research.135681.3 (PMC11358685; doi:10.12688/f1000research.135681.3)
Supplement: Peer review history

# Reviewer report: Reviewer response for version 3

**DOI:** 10.5256/f1000research.167059.r314188

**Published:** 2024-08-28

**Authors:** Natale Calomino

**Affiliations:** 1 University of Siena, Siena, Italy

The aim of the work is to investigate the possibility of predicting surgical site infection. The abstract induces the curiosity to read the article in its entirety. in the introduction we focus on the fact that surgical site infection is nosocomial. This concept needs to be described better, in fact it is nosocomial because it happens in hospital, or it is nosocomial because the contaminating bacteria are of nosocomial origin, for example we can notice that in a smaller ward the patients have wounds infected by the same bacterial strains. We absolutely agree on the factors that induce infections. The study design and methods are well orchestrated with the right inclusion and exclusion criteria. We also agree on the approval of the ethics committee. We learn without having to add anything about the results. In the discussion we absolutely agree on what was said regarding the socio-economic factors and the clinical conditions with which patients present themselves in hospital. I liked what was said about hair removal, to this I would add the shower before each operation and antibiotic prophylaxis, in this regard I recommend an article already the subject of numerous citations [ref 1 ], to be cited in the bibliography. We agree on the conclusions. Great digression on limitations. English worth reviewing, good iconography, even if the images are a bit small. The bibliography is a good basis for the article.

Is the work clearly and accurately presented and does it cite the current literature?

Yes

If applicable, is the statistical analysis and its interpretation appropriate?

Yes

Are all the source data underlying the results available to ensure full reproducibility?

Yes

Is the study design appropriate and is the work technically sound?

Yes

Are the conclusions drawn adequately supported by the results?

Yes

Are sufficient details of methods and analysis provided to allow replication by others?

Yes

Reviewer Expertise:

generale surgery, oncological surgery

I confirm that I have read this submission and believe that I have an appropriate level of expertise to confirm that it is of an acceptable scientific standard, however I have significant reservations, as outlined above.

## References

[1] : Antimicrobial Prophylaxis Reduces the Rate of Surgical Site Infection in Upper Gastrointestinal Surgery: A Systematic Review. *Antibiotics (Basel)* .2022;11(2) : 10.3390/antibiotics11020230 10.3390/antibiotics11020230 35203832 PMC8868284

# Reviewer report: Reviewer response for version 2

**DOI:** 10.5256/f1000research.161396.r258951

**Published:** 2024-04-26

**Authors:** Prakash Kumar Sasmal

**Affiliations:** 1 AIIMS, Bhubaneswar, India

Dear authors,

 This retrospective study aims to determine prediction factors for surgical site infection in a developing country after gastrointestinal surgery. SSIs are very common and distressing not only to the patients but also to the surgeons. Time and again, it is clear that SSIs after surgery are caused by several factors and not a single factor correction will ever decrease the rate. However, a few points need to be clarified to understand the readers better, as recall bias is more expected in a retrospective study. As multiple variables are taken to correlate only the associations, it becomes challenging to point out a particular cause to rectify.

 1- As far as I can interpret in the study, the authors have put leucocytosis and high neutrophils as one of the factors for SSIs. Is leucocytosis the cause or effect of SSIs?

 2- The prolonged hospital stays >5 days were the cause of SSIs or the effect of the infection, for which the patients were not discharged.

 3- The Khat chewing, as mentioned in Table 5, has spelling errors.

 4- The type of organ-specific SSIs in case of bowel, biliary and pancreatic surgery need to be specified whether there was a documented leak in those cases. Especially in pancreatic cases, whether preop stenting was done or not.

 5- The organisms responsible need to be subclassified according to the bowel, biliary or pancreatic surgery, as the readers will benefit from knowing the common bacteria involved.

 6- The authors have tried to correlate the incidence of SSIs in males and females. I do not think comparing the incidences of various genders is prudent, as it is a matter of chance.

 7- The approach of surgery, whether open or laparoscopic, should be well mentioned as the details can be easily retrieved from the data. We cannot compare open surgery incidences of SSIs with laparoscopic surgery.

 8- The common pathogens isolated were E coli and *Enterococcus*. The authors need to mention facts about the sterilisation and disinfection techniques used in your hospital to rule out a common source.

 9- There is a repetition of the reference No 13 and 14. Please check it.

 10- The authors need to put a few lines based on studies regarding steps to reduce SSIs overall in gastrointestinal procedures.

Is the work clearly and accurately presented and does it cite the current literature?

Partly

If applicable, is the statistical analysis and its interpretation appropriate?

Yes

Are all the source data underlying the results available to ensure full reproducibility?

Yes

Is the study design appropriate and is the work technically sound?

Partly

Are the conclusions drawn adequately supported by the results?

Yes

Are sufficient details of methods and analysis provided to allow replication by others?

Yes

Reviewer Expertise:

Gastrointestinal and Metabolic surgery

I confirm that I have read this submission and believe that I have an appropriate level of expertise to confirm that it is of an acceptable scientific standard, however I have significant reservations, as outlined above.

# Response

**Published:** 2024-05-25

**Authors:** Faisal Ahmed

APPROVED WITH RESERVATIONS

 Dear authors,

 This retrospective study aims to determine prediction factors for surgical site infection in a developing country after gastrointestinal surgery. SSIs are very common and distressing not only to the patients but also to the surgeons. Time and again, it is clear that SSIs after surgery are caused by several factors, and not a single factor correction will ever decrease the rate. However, a few points need to be clarified to understand the readers better, as recall bias is more expected in a retrospective study. As multiple variables are taken to correlate only the associations, it becomes challenging to point out a particular cause to rectify.

  

1. As far as I can interpret in the study, the authors have put leucocytosis and high neutrophils as one of the factors for SSIs. Is leucocytosis the cause or effect of SSIs?
**Answer:** Thank you very much.
  Leukocytosis was a predictive factor for SSI occurrence and not the cause.
2. The prolonged hospital stays >5 days were the cause of SSIs or the effect of the       infection, for which the patients were not discharged.
**Answer:** Thank you very much.
  Prolonged hospital stays >5 days were a predictive factor for SSI occurrence and not the cause.
3. The Khat chewing, as mentioned in Table 5, has spelling errors.
**Answer:** Thank you very much.
  It was revised as you mentioned.
4.  The type of organ-specific SSIs in case of bowel, biliary, and pancreatic surgery need to be specified whether there was a documented leak in those cases. Especially in pancreatic cases, whether preop stenting was done or not.
** Answer:** Thank you very much.
  Unfortunately, the data on postoperative leaks is not available for all cases. a future study focused on SSI occurrence and specific postoperative complications in specific organs is planned.
5. The organisms responsible need to be subclassified according to the bowel, biliary, or pancreatic surgery, as the readers will benefit from knowing the common bacteria involved.
**Answer:** Thank you very much.
  It was revised and mentioned in Table 3.
6. The authors have tried to correlate the incidence of SSIs in males and females. I do not think comparing the incidences of various genders is prudent, as it is a matter of chance.
**Answer:** Thank you very much.
**This factor was not statistically significant.** Additionally, we could not remove any collected factor without justification.
7. The approach of surgery, whether open or laparoscopic, should be well mentioned as the details can be easily retrieved from the data. We cannot compare open surgery incidences of SSIs with laparoscopic surgery.
**Answer:** Thank you very much.
  Unfortunately, we did not have laparoscopic equipment in our center.
8. The common pathogens isolated were E coli and Enterococcus. The authors need to mention facts about the sterilization and disinfection techniques used in your hospital to rule out a common source.
**Answer:** Thank you very much.
  We added a paragraph on this issue in the discussion section as you recommended in the microorganisms responsible for SSIs paragraph. The added section was " Additionally, strict adherence to surgical site infection prevention techniques Such as disinfection and sterilization of medical and surgical tools to avoid the spread of infectious germs need to get more attention. Healthcare rules should specify whether cleaning, disinfection, or sterilization is required based on the item's intended usage".
9. There is a repetition of the reference No 13 and 14. Please check it.
** Answer:** Thank you very much.
  The reference 13 was changed as you recommended.
10. The authors need to put a few lines based on studies regarding steps to reduce SSIs overall in gastrointestinal procedures
**Answer:** Thank you very much.
**We added a paragraph in the discussion section regarding this issue.** The added section is " Surgical site infections can be prevented by a variety of techniques, including improved preoperative surgical site preparation, good infection control management during procedures, careful adherence to prophylactic antibiotics administration, and a variety of preventive measures aimed at neutralizing the threat of bacterial, viral, and fungal contamination posed by operative staff, the operating room environment, and the patient's endogenous skin flora. Glucose-level control, improved oxygen supply, and normothermia maintenance are three new areas that have the potential to lower the incidence of SSIs even further. Continuous study into the biology of SSIs, as well as rigorous adherence to the use of evidence-based proven techniques to minimize SSIs, can help to further reduce the health and cost repercussions of SSIs."

 

1. Is the work clearly and accurately presented and does it cite the current literature?

-
  - Partly
  - **Answer:** Thank you very much.
  - All mentioned comments were responded to and included in the revised manuscript.

 

1. Is the study design appropriate and is the work technically sound?

-
  - Partly
  - **Answer:** Thank you very much. All mentioned comments were responded to and included in the revised manuscript.

 

1. Are sufficient details of methods and analysis provided to allow replication by others?

-
  - Yes
  - **Answer:** Thank you very much.

 

1. If applicable, is the statistical analysis and its interpretation appropriate?

-
  - Yes
  - **Answer:** Thank you very much.

 

1. Are all the source data underlying the results available to ensure full reproducibility?

-
  - Yes
  - **Answer:** Thank you very much.

 

1. Are the conclusions drawn adequately supported by the results?

-
  - Yes
  - **Answer:** Thank you very much.

 

1. Competing Interests
  - No competing interests were disclosed.
  - **Answer:** Thank you very much.

 

1. Reviewer Expertise
2. Gastrointestinal and Metabolic surgery

 

-
  - I confirm that I have read this submission and believe that I have an appropriate level of expertise to confirm that it is of an acceptable scientific standard, however, I have significant reservations, as outlined above.
  - **Answer:** Thank you very much.

# Reviewer report: Reviewer response for version 2

**DOI:** 10.5256/f1000research.161396.r238885

**Published:** 2024-02-07

**Authors:** Nicolas Troillet

**Affiliations:** 1 Department for Infectious Diseases, Central Institution, Valais Hospital, Sion, Switzerland

No new comment. The article has been substantially improved by the authors' revision

Is the work clearly and accurately presented and does it cite the current literature?

Yes

If applicable, is the statistical analysis and its interpretation appropriate?

Partly

Are all the source data underlying the results available to ensure full reproducibility?

Yes

Is the study design appropriate and is the work technically sound?

Yes

Are the conclusions drawn adequately supported by the results?

Yes

Are sufficient details of methods and analysis provided to allow replication by others?

Partly

Reviewer Expertise:

Healthcare associated infections, clinical infectious diseases.

I confirm that I have read this submission and believe that I have an appropriate level of expertise to confirm that it is of an acceptable scientific standard.

## References

[1] : Identification of predictive factors for surgical site infections in gastrointestinal surgeries: A retrospective cross-sectional study in a resource-limited setting. *F1000Research* .2024;12: 10.12688/f1000research.135681.2 10.12688/f1000research.135681.2 PMC1135868539211056

# Response

**Published:** 2024-02-08

**Authors:** Faisal Ahmed

Dear Reviewer

 Thank you very much for your effort in acting on my revised manuscript.

# Reviewer report: Reviewer response for version 1

**DOI:** 10.5256/f1000research.148812.r192612

**Published:** 2023-08-25

**Authors:** Nicolas Troillet

**Affiliations:** 1 Department for Infectious Diseases, Central Institution, Valais Hospital, Sion, Switzerland

This is an interesting and well written study aiming at determining a prediction rule for surgical site infection after digestive surgery in a developing country. It gathered a substantial number of parameters for univariate and multivariable analysis in order to develop a prediction model that performs better than the classical NNIS index.

 The authors might want to consider the comments and questions below and revise their paper accordingly.

  

1. The abstract mentions a SSI rate of 15% (79/525), whereas the results section and the discussion state 16.4% (86/525).
2. The abstract states that the majority of SSI were superficial but detailed results on the depth of SSI (superficial, deep, organ/space) are not provided in the results section. These data would be interesting by type of surgery.
3. The inclusion criteria state that all eligible patients between June 2015 and October 2022 were included, reaching 525 patients in about 7 years. Knowing the number of patients excluded would be of interest.
4. Precisions on how SSI were detected would be of interest. Was the diagnosis made by surgeons or infection control personnel? Were all the patients followed-up after discharge? If yes, by systematic visits? All SSI were microbiologically documented. Was it because samples were taken from all patients with a clinically diagnosed SSI or because positive lab results were used to identify patients with SSI?
5. Operations are grouped in four categories (small bowel, large bowel, biliary, and pancreatic) in the description of the population analyzed. More details would be interesting to know which operations were included in these four categories, e.g. how many cholecystectomies were included in biliary surgery? Were appendectomies included in large bowel surgery or only colon surgery? Did large bowel surgery include rectal surgery?
6. These operations are then grouped in two categories (small bowel and others) in the statistical analyses. Although small bowel surgery represents the highest number of included operations, grouping large bowel surgery with biliary surgery appears counterintuitive, especially if cholecystectomies, which are much less at risk than colon surgery, represent the majority of biliary surgery. Could the results have been different and reached statistical significance if the comparison had been made between large bowel and others rather than between small bowel and others?
7. Some continuous variables such as age, hospital stay, BMI or temperature were stratified into binomial variables for the statistical analyses. Did it imply a loss of power for detecting risk factors and perhaps better determine a cut-off for the prediction? This applies particularly for age which could have been considered as an ordinal variable and stratified first in ten-year categories.
8. The timing of shaving was found significant but shaving is not recommended by international guidelines and may constitute a risk factor for SSI (cf. for example reference 2 in the present paper). Is shaving systematically done in this hospital? Avoiding it accordingly with guidelines could constitute a simple mean for decreasing SSI rates that could be mentioned in the discussion.
9. It is stated that variables with a p value <0.2 in univariate analysis were fitted for logistic regression but “antibiotic timing” and “operative type”, which had both higher p values in univariate analysis (p=0.454 and p=0.506, respectively, as presented in table 4) are nevertheless part of the multivariable model (table 5). More details would be useful for a better understanding.
10. Do all the operations performed in digestive surgery in this hospital correspond to open surgery? Since operations done with a laparoscope have been shown to be less at risk for SSI, information would be of interest about it in the methods and in the discussion.
11. It is stated that “pathogens linked with SSI were identified from all SSI patient wounds”. The total number of pathogens in table 3 amounts to 86 for 86 patients. This would mean that only one pathogen was identified from every patient with SSI. Were no patients suffering from a polymicrobial SSI detected (which is relatively frequent in colon surgery)? Were no other pathogens identified than the seven presented in table 3? For example, Enterobacter spp. streptococci or Candida spp.?
12. More details would be of interest on resistance patterns of the isolated bacteria: proportion of ESBL producers in E. coli and Klebsiella respectively, presence or not of carbapenemase-producing Enterobacteriaceae, resistance profile of Pseudomonas aeruginosa, resistance to vancomycine in enterococci.
13. Mentioning the substances usually administered for antibiotic prophylaxis in these operations would help better realizing the magnitude of the problem of antibiotic resistance in this setting.
14. Were only the significant variables in the multivariable analysis used for calculating the performance of the model for predicting SSI? Please specify.
15. Discussion, paragraph 6. Mentioning whether glucose levels were not available for the included patients together with albumin levels or were available but not analyzed would be of interest since, irrespective of a history of diabetes, this could constitute, as stated, a risk factor for SSI.
16. Discussion, paragraph 9. The following sentence is hard to understand: “Furthermore, our study revealed that the prevalence of SSI in large, pancreatic, and biliary surgeries was lower than in intestinal procedures; although this association was not statistically significant.” Does “large” mean large bowel? Then “intestinal” might mean small bowel. Please clarify. In addition, please refer to comments 5 and 6 above for possible modifications in the discussion about this result.
17. Discussion, paragraph 10. It is stated that a long duration may induce weaning prophylactic antibiotic concentration. Studies have shown the importance of re-dosing when this duration reach the half-life of the administered antibiotic and guidelines recommend it. This could be mentioned here and cited as a mean to lower SSI rates.
18. Discussion, paragraph 11. Please refer to comments 11, 12, and 13 above and possibly take them into account for extending the discussion about antibiotic resistance in this paragraph.
19. Limitations, 3 ^rd^ sentence. Does “clinical identification” mean that no radiological or laboratory markers were used to help for the diagnosis of SSI? If yes, this should be specified in the methods. If not, underreporting might be due to interobserver variability if persons with different backgrounds did it (e.g. surgeons, infectious diseases physicians, infection control nurses).
20. Limitations, 4 ^th^ sentence. Internal and external validation on other datasets or prospectively should be mentioned as a mean to consolidate these findings.
21. Typos. Table 1: “During 1 hour of operation” instead of “During 1 house of operation”. Table 3: “Pseudomonas aeruginosa” instead of “Pseudomonas aerugisa”.
22. Terminology. The same words should be used through the different tables (e.g. “surgical status” in table 1 instead of “operative type” in tables 4 and 5). Consider using “operation duration” instead of “operative time”.
23. References 13 and 14 are identical. Reference 18 corresponds to a correction: please mention the original publication.

(The source data are available but the corresponding file could not be opened.)

Is the work clearly and accurately presented and does it cite the current literature?

Yes

If applicable, is the statistical analysis and its interpretation appropriate?

Partly

Are all the source data underlying the results available to ensure full reproducibility?

Yes

Is the study design appropriate and is the work technically sound?

Yes

Are the conclusions drawn adequately supported by the results?

Yes

Are sufficient details of methods and analysis provided to allow replication by others?

Partly

Reviewer Expertise:

Healthcare associated infections, clinical infectious diseases.

I confirm that I have read this submission and believe that I have an appropriate level of expertise to confirm that it is of an acceptable scientific standard, however I have significant reservations, as outlined above.

# Response

**Published:** 2024-01-25

**Authors:** Faisal Ahmed

Dear reviewer

 Thank you very much for reading and reviewing my manuscript.

 This is an interesting and well-written study aiming at determining a prediction rule for surgical site infection after digestive surgery in a developing country. It gathered a substantial number of parameters for univariate and multivariable analysis to develop a prediction model that performs better than the classical NNIS index.

 The authors might want to consider the comments and questions below and revise their paper accordingly.

1. The abstract mentions an SSI rate of 15% (79/525), whereas the results section and the discussion state 16.4%. (86/525)

**Answer: **Thank you.

 We apologize for this mistake. The exact number was revised in the abstract section.

1. The abstract states that the majority of SSI were superficial but detailed results on the depth of SSI (superficial, deep, organ/space) are not provided in the results section. These data would be interesting by type of surgery.

**Answer: **Thank you.

  The SSI type was added to the result section (Causative Pathogens section).

1. The inclusion criteria state that all eligible patients between June 2015 and October 2022 were included, reaching 525 patients in about 7 years. Knowing the number of patients excluded would be of interest.

**Answer:** Thank you.

 Unfortunately, the total number of patients was not accurately calculated. For that, we avoided mentioning the total patient numbers.

1. Precisions on how SSIs were detected would be of interest. Was the diagnosis made by surgeons and infection control personnel? Were all the patients followed up after discharge? If yes, by systematic visits? All SSIs were microbiologically documented. Was it because samples were taken from all patients with a clinically diagnosed SSI or because positive lab results were used to identify patients with SSI?

**Answer: **Thank you.

 Regarding infection control personnel, it was corrected.

 Regarding patients followed up: yes. As they were operated, it is usually to have a regular follow-up. Additionally, we mentioned that they underwent systematic visits. Regarding microbiology documentation, all patients had culture and we mentioned it in the data collection section. The main causes of sample collection are the patient symptoms and the SSI suspected.

1. Operations are grouped into four categories (small bowel, large bowel, biliary, and pancreatic) in the description of the population analyzed. More details would be interesting to know which operations were included in these four categories, e.g. how many cholecystectomies were included in biliary surgery? Were appendectomies included in large bowel surgery or only colon surgery? Did large bowel surgery include rectal surgery?

**Answer: **Thank you.

 We did not collect the data regarding

1. These operations are then grouped into two categories (small bowel and others) in the statistical analyses. Although small bowel surgery represents the highest number of included operations, grouping large bowel surgery with biliary surgery appears counterintuitive, especially if cholecystectomies, which are much less at risk than colon surgery, represent the majority of biliary surgery. Could the results have been different and reached statistical significance if the comparison had been made between Large bowel and others rather than between small bowel and others?

**Answer: **Thank you.

 We revised it as you mentioned. However, it was not statistically significant as we mentioned in table 4.

 Some continuous variables such as age, hospital stay, BMI or temperature were stratified into binomial variables for the statistical analyses. Did it imply a loss of power for detecting risk factors and perhaps better determine a cut-off for the prediction? This applies particularly for age which could have been considered as an ordinal variable and stratified first in ten-year categories.

 Answer: Thank you.

 All the continuous variables were converted into categorical variables for a better presentation of the nomogram. This statement was mentioned.

  

1. The timing of shaving was found significant but shaving is not recommended by international guidelines and may constitute a risk factor for SSI (cf. for example reference 2 in the present paper). Is shaving systematically done in this hospital? Avoiding it according with guidelines could constitute a simple means for decreasing SSI rates that could be mentioned in the discussion.

Answer: Thank you.

 This item was revised to be Hair removal. We mentioned this issue in the discussion section.

1. It is stated that variables with a p value <0.2 in univariate analysis were fitted for logistic regression but “antibiotic timing” and “operative type”, which had both higher p values in univariate analysis (p=0.454 and p=0.506, respectively, as presented in table 4) are nevertheless part of the multivariable model (table 5). More details would be useful for a better understanding.

Answer: Thank you.

 To better understand, we revised the Statistical analysis section.

 10.       Do all the operations performed in digestive surgery in this hospital correspond to open surgery? Since operations done with a laparoscope have been shown to be less at risk for SSI, information would be of interest about it in the methods and in the discussion.

 Answer: Thank you.

 Unfortunately, the laparoscopic equipment is not available in our hospital. For that, we avoided mentioning the laparoscopic procedures.

 11.       It is stated that “pathogens linked with SSI were identified from all SSI patient wounds”. The total number of pathogens in table 3 amounts to 86 for 86 patients. This would mean that only one pathogen was identified from every patient with SSI. Were no patients suffering from a polymicrobial SSI detected (which is relatively frequent in colon surgery)? Were no other pathogens identified than the seven presented in Table 3? For example, Enterobacter spp. streptococci or Candida spp.?

 Answer: Thank you.

 This was the result reported by the laboratory.

 12.       More details would be of interest on resistance patterns of the isolated bacteria: proportion of ESBL producers in E. coli and Klebsiella respectively, presence or not of carbapenemase-producing Enterobacteriaceae, resistance profile of Pseudomonas aeruginosa, resistance to vancomycin in enterococci.

 Answer: Thank you.

 We did not collect the data regarding the antibiotic sensitivity or resistance.

 13.       Mentioning the substances usually administered for antibiotic prophylaxis in these operations would help better realize the magnitude of the problem of antibiotic resistance in this setting.

 Answer: Thank you. it was mentioned in the data collection section.

 14. Were only the significant variables in the multivariable analysis used for calculating the performance of the model for predicting SSI? Please specify

 . Answer: Thank you.

 To better understand, we revised the Statistical analysis section.

 15.       Discussion, paragraph 6. Mentioning whether glucose levels were not available for the included patients together with albumin levels or were available but not analyzed would be of interest since, irrespective of a history of diabetes, this could constitute, as stated, a risk factor for SSI.

 Answer: Thank you.

 We mentioned this issue in the discussion section.

 16.       Discussion, paragraph 9. The following sentence is hard to understand: “Furthermore, our study revealed that the prevalence of SSI in large, pancreatic, and biliary surgeries was lower than in intestinal procedures; although this association was not statistically significant.” Does “large” mean large bowel? Then “intestinal” might mean small bowel. Please clarify. In addition, please refer to comments 5 and 6 above for possible modifications in the discussion about this result.

 Answer: Thank you.

 It was revised as you mentioned.

  17.      Discussion, paragraph 10. It is stated that a long duration may induce weaning prophylactic antibiotic concentration. Studies have shown the importance of re-dosing when this duration reaches the half-life of the administered antibiotic and guidelines recommend it. This could be mentioned here and cited as a means to lower SSI rates.

 Answer: Thank you.

 It was added as you mentioned.

 18.       Discussion, paragraph 11. Please refer to comments 11, 12, and 13 above and possibly take them into account for extending the discussion about antibiotic resistance in this paragraph.

 Answer: Thank you.

 We did not collect the data regarding the antibiotic sensitivity or resistance.

 19.       Limitations, 3rd sentence. Does “clinical identification” mean that no radiological or laboratory markers were used to help with the diagnosis of SSI? If yes, this should be specified in the methods. If not, underreporting might be due to interobserver variability if persons with different backgrounds did it (e.g. surgeons, infectious diseases physicians, infection control nurses).

 Answer: Thank you.

 The word clinical was removed.

 20.       Limitations, 4th sentence. Internal and external validation on other datasets or prospectively should be mentioned as a mean to consolidate these findings.

 Answer: Thank you.

 We added this issue as you recommended.

 21.       Typos. Table 1: “During 1 hour of operation” instead of “During 1 house of operation”. Table 3: “Pseudomonas aeruginosa” instead of “Pseudomonas aerugisa”.

 Answer: Thank you.

 It was revised through the manuscript.

 22.       Terminology. The same words should be used through the different tables (e.g. “surgical status” in Table 1 instead of “operative type” in Tables 4 and 5). Consider using “operation duration” instead of “operative time”.

  Answer: Thank you.

 It was revised as you mentioned.

 23.       References 13 and 14 are identical. Reference 18 corresponds to a correction: please mention the original publication.

 Answer: Thank you.

 All references were rechecked and corrected

 The source data are available but the corresponding file could not be opened.

 Answer: Thank you.

 The revised dates are available at this address.

https://data.mendeley.com/datasets/hk75wrwr6n/2

             Is the work clearly and accurately presented and does it cite the current literature?

 Yes

 Answer: Thank you.

             Is the study design appropriate and is the work technically sound?

 Yes

 Answer: Thank you.

             Are sufficient details of methods and analysis provided to allow replication by others?

 Partly

 Answer: Thank you.

             If applicable, is the statistical analysis and its interpretation appropriate?

 Partly

 Answer: Thank you.

             Are all the source data underlying the results available to ensure full reproducibility?

 Yes

 Answer: Thank you.

             Are the conclusions drawn adequately supported by the results?

 Yes

 Answer: Thank you.

 Competing Interests

 No competing interests were disclosed.

 Answer: Thank you.

 Reviewer Expertise

 Healthcare associated infections, clinical infectious diseases.

 Answer: Thank you.
